# Melatonin Uptake by Cells: An Answer to Its Relationship with Glucose?

**DOI:** 10.3390/molecules23081999

**Published:** 2018-08-10

**Authors:** Juan C. Mayo, Arturo Aguado, Rafael Cernuda-Cernuda, Alejandro Álvarez-Artime, Vanesa Cepas, Isabel Quirós-González, David Hevia, Rosa M. Sáinz

**Affiliations:** 1 Departamento de Morfología y Biología Celular, Facultad de Medicina, Universidad de Oviedo, 33006 Oviedo, Spain; arturoaguadog@gmail.com (A.A.); rcernuda@uniovi.es (R.C.-C.); alejandroalvarezartime@gmail.com (A.Á.A.); cepasvanesa@uniovi.es (V.C.); quirosisabel@uniovi.es (I.Q.-G.); heviadavid@uniovi.es (D.H.), sainzrosa@uniovi.es (R.M.S.); 2 Instituto Universitario Oncológico del Principado de Asturias, Universidad de Oviedo; 33006 Oviedo, Spain

**Keywords:** melatonin, glucose, GLUT/SLC2A, metabolism, insulin

## Abstract

Melatonin, *N*-acetyl-5-methoxytryptamine, is an indole mainly synthesized from tryptophan in the pineal gland and secreted exclusively during the night in all the animals reported to date. While the pineal gland is the major source responsible for this night rise, it is not at all the exclusive production site and many other tissues and organs produce melatonin as well. Likewise, melatonin is not restricted to vertebrates, as its presence has been reported in almost all the phyla from protozoa to mammals. Melatonin displays a large set of functions including adaptation to light: dark cycles, free radical scavenging ability, antioxidant enzyme modulation, immunomodulatory actions or differentiation–proliferation regulatory effects, among others. However, in addition to those important functions, this evolutionary ‘ancient’ molecule still hides further tools with important cellular implications. The major goal of the present review is to discuss the data and experiments that have addressed the relationship between the indole and glucose. Classically, the pineal gland and a pinealectomy were associated with glucose homeostasis even before melatonin was chemically isolated. Numerous reports have provided the molecular components underlying the regulatory actions of melatonin on insulin secretion in pancreatic beta-cells, mainly involving membrane receptors MTNR1A/B, which would be partially responsible for the circadian rhythmicity of insulin in the organism. More recently, a new line of evidence has shown that glucose transporters GLUT/SLC2A are linked to melatonin uptake and its cellular internalization. Beside its binding to membrane receptors, melatonin transportation into the cytoplasm, required for its free radical scavenging abilities, still generates a great deal of debate. Thus, GLUT transporters might constitute at least one of the keys to explain the relationship between glucose and melatonin. These and other potential mechanisms responsible for such interaction are also discussed here.

## 1. Melatonin: A Universal and Ubiquitous Molecule

Melatonin, chemically *N*-acetyl-5-methoxytryptamine, was first isolated and further characterized by Lerner and co-workers in the late 1950s [1,2]. However, 40 years before, in 1917, McCord and Allen reported for the first time the ability of cow pineal extracts to lighten tadpoles when they were fed with them [3]. This clue was further exploited by Lerner and co-workers who coined the name ‘melatonin’ for this substance, a portmanteu from ‘mela’ (able to lighten) and ‘tonin’ because of its derived from serotonin. As a consequence, this could be considered the first, and yet minor, function atributed to the indole. Two years later, Axelrod and Weissbach identified the rate limiting synthetic enzymes, namely *N*-acetyl-transferase (aralkylamine-NAT, AANAT) and hydroxyindole-*O*-methyl-transferase (acetylserotonin *O*-methyl transferase or ASMT/HIOMT), which control melatonin synthesis from tryptophan in the pineal [4]. This epithalamic gland is responsible for melatonin nocturnal serum rise, while light signals, transduced by the retina and transmitted via suprachiasmatic nuclei, constitute its major synthesis inhibitor [5,6]. Due to its association with the day: night cycle melatonin was rapidly associated with the regulation of circadian rhythms and further shown to modulate reproduction in seasonal breeding animals by adapting their physiology to external photoperiodic conditions [7,8], an evolutionarily acquired mechanism that ensures offspring survival [9,10]. Duration and intensity of light regulate melatonin production [11,12] and nocturnal production decays with age [13,14]. This fact prompted the further investigation of melatonin effects on aging and neurodegeneration [15,16,17,18]. In addition to this important function, it has also been linked to other important roles including immunomodulation or anti-cancer actions [19,20,21,22,23,24].

Melatonin production was initially thought to be restricted to the pineal gland, but pinealectomies did not completely remove it in rodents. Highly sensitive detection methods revealed that about 20% of normal levels was still present in serum or urine, although these basal levels were not modified by light–dark rhythms [25]. In 1965, Quay first showed in a pioneer study the existence of HIOMT (now ASMT) in both retina and pineal gland and, later on, the same activity was also demonstrated in the orbital Harderian gland [26]. Years later, Bubenik’s and Kvetnoy’s groups were the first to show that melatonin can be localized outside the pineal gland [27,28]. Thus, retina, cerebellum and the enterochromaffin cells, the latter as part of the APUD system (now commonly referred to as ‘diffuse endocrine system’), were the first organs/cells in which authors showed the potential local production of melatonin. Soon after that, the Harderian gland was also found to demonstrate an immunopositive reaction to melatonin [29], thus corroborating the findings mentioned above. However, in all these studies the demonstration included non-analytical detection strategies, but rather was based on the immunohistochemical detection of either synthetic enzymes (AANAT/ASMT) or melatonin itself. Even though this methodology could generate some doubts, these first findings definitively boosted the search for new sites of melatonin synthesis. The introduction of radioimmunoassay (RIA) [30] was necessary to reinforce evidence about the presence of extra-pineal melatonin, particularly after the discovery of ^125^I-radiolabeled melatonin as a tracer [31,32]. Not only did this technique allow the quantification of serum melatonin, but it also allowed the discovery of high-affinity melatonin-binding sites in rat synaptosomal preparations [33], as well as in the brain of other species, including human suprachiasmatic nuclei [34,35,36]. 

Thanks to the use of RIA and more recently to high-performance liquid chromatography (HPLC) [30,37], the production of the indole has been accurately assayed in several tissues and organs including the retina, Harderian glands, gut, testis or skin, among many others, but these local productions do not seem to contribute to the nocturnal serum levels [38]. The extra-pineal sites of melatonin production and the corresponding references are summarized in Table 1. Among these extracellular sites of melatonin production, one of the first organs reported in which melatonin is thought to play a local key function was the retina [27]. Additionally, two other sites of production appear to be of particular importance, i.e., the gut and the skin, due to the relatively high amount and also the key physiological function played [39,40,41,42]. At least in the skin, melatonin synthesis can take a shortcut through an AANAT-independent pathway involving alternative enzymes [43]. 

In addition to the ubiquitous presence of melatonin in so many tissues, the indole has been identified in evolutionarily different organisms. One of the breakthrough discoveries in this field was made by Hardeland’s group, who first discovered the indole as well as its synthesis in the dinoflagellate *Lyngulodinium polyedrum* (syn *Gonyaulax polyedra*) [44]. Melatonin appears to be present at least in some bacteria (*Rodospirillum* rubrum) [45], unicellular phyla [46,47] as well as in *Saccharomyces cerevisiae* [48]. Likewise, the presence of melatonin in Arthropoda, Nematoda, nemertine worms or Gastropoda, among others, have also been shown [49,50,51,52,53]. More surprisingly, the discovery in fungi [53] and in vascular plants [54,55] has now attracted the attention of researchers to many other groups, and melatonin has been found in red and brown algae (Rhodophyta, Phaeophyta), green algae and land plants (Viridiplantae) [56,57,58,59]. This brings out the question of the ancient and basic role(s) that melatonin seems to play in all these organisms, likely preceding the more “modern” circadian-related actions [57,60]. The presence of melatonin in the different phyla of invertebrates is shown in Table 2.

The antioxidant properties of melatonin, discovered in the early 1990s [104] has opened a new research line that has highlighted the important cytoprotective actions of the indole in almost every single organ, tissue and cell type [105], with important clinical implications in neurodegeneration, cancer or ischemia-reperfusion related pathologies. Similarly, melatonin might also show protection against stress-related challenges in plants [43]. Melatonin reaction with free radicals give different metabolites, namely cyclic 3-OH-melatonin, *N*(1)-acetyl-*N*(2)-formyl-5-methoxykynuramine (AFMK) and *N*(1)-acetyl-5-methoxykynuramine (AMK), which also show free radical-scavenging activities, thus converting melatonin in a very efficient free radical chain reaction [43]. These melatonin metabolite reactions might be of importance in protecting those tissues/organs that act as barriers in the organism, e.g., skin or gut, where melatonin is produced in significant quantitites [106]. 

## 2. Vesicle Secretion or Membrane Diffusion?

### 2.1. Vesicles in the Pineal Gland

The pineal organ of non-mammalian vertebrates is photoreceptive and pinealocytes exhibit a very similar morphology to that of retinal photoreceptors. These cells display an outer segment containing multiple membrane discs, an intermediate part with a ‘9 + 0’ cilium structure, an inner segment, and a basal part with basal processes. This basal part usually contains multiple secretory vesicles [107]. The outer photoreceptor-like segment become partially lost in the mammalian pinealocytes, thus leading to the lack of photoreceptive function. However, while these pinealocytes evolved toward a non-sensory structure, they also show numerous vesicles, frequently associated to synaptic ribbons or rosette-like structures, indicating high secretory function [108]. There is a controversy about the exact content of such vesicles. Some of them are also known as Golgi dense-core vesicles (DCR) while others show a more classic homogeneous electro-lucent content. The number of vesicles are subjected to opposite circadian rhythmicity [109] and it is altered by either superior cervical gangliectomy or melatonin treatment itself [110]. Interestingly, some authors have reported that DCR contain neither melatonin nor serotonin (5 hydroxy-tryptamine, 5-HT) and the electron-dense material observed within DCR would rather correspond to pineal neuropeptides [108,111]. On the contrary, Juillard and Collin [112], based on fluorescence histochemical methods, determined that 5-HT fluorescence corresponded with DCV. The pinealocytes can, therefore, be considered part of the diffuse NE system, as the secretion products are released either into the pineal recess blood vessels or even directly into the CVF at the third ventricle [113]. Both serotonin and melatonin display important regulating functions on their own secretion. Serotonin itself, through 5-HT2 receptors, may contribute to the optimal secretion of melatonin, as has been shown in cultured pinealocytes [114,115]. This would explain why serotonin always precedes a melatonin peak during the dark phase in all the species studied [114]. A similar role has been found for glutamate, which also seems to accumulate inside the pinealocyte vesicles [116]. More importantly, this would indicate that these paracrine/autocrine mediators would modulate the release of melatonin, thus indicating a more complex regulatory mechanism than simple diffusion, which depends exclusively on the relative concentrations at both sides of the membranes, must also be involved. Nevertheless, the coexistence of diffusion through cell membranes with other transporter or carrier-like systems should not be completely discarded.

### 2.2. Chemical Features of Melatonin and Membrane Diffusion

According to PubChem database (all the chemical information has been consulted at https://pubchem.ncbi.nlm.gov), melatonin solubility in ethanol is very high (182 g/L) while the solubility in water varies from roughly 2 g/L (20 °C) to 3.5 g/L, a feature confirmed by Shida and co-workers, who were able to solubilize melatonin at 5 mM [117]. Strictly from the chemical point of view, this should be considered a moderate to high hydro-solubility and, more importantly, this would make unnecessary the involvement of serum proteins as blood carriers for the indole. Contrary to this hypothesis, Li and Wang have recently reported that melatonin binds to human serum albumin in a 1:1 stoichiometry [118]. As a reference, serotonin solubility in water is 10-fold higher (>25 g/L) [118]. As a reference, serotonin’s solubility in water is 10-fold higher (>25 g/L) while a cholesterol-derived steroid such as testosterone is much lower (33 mg/L). This high testosterone lipophilicity makes these substances thermodynamically compatible with rapid lipid bilayer diffusion [119]. Like the rest of the steroid hormones, testosterone is supposed to be continuously liberated through the lipid bilayer in a gradient-favored manner. Interestingly, its release, which is finely regulated by LH in Leydig cells, might involve additional secretory mechanisms. Likewise, the same principle would apply to melatonin, which is thought to be released by diffusion, but it is under the control of different stimuli (see above). Nevertheless, considering the relative indole lipophilicity, the diffusion rate should be rather low when compared to androgens or estrogens. The question remains as to how much of this membrane diffusion has been really investigated throughout the literature. Surprisingly, the answer is that not so many studies have been focused on this issue.

### 2.3. Melatonin and Interactions with Lipid Membranes 

For many years, most authors have assumed that melatonin, due to some of the physical features mentioned above, moves across biological membranes through passive diffusion. Additionally, the ubiquity of melatonin’s actions, the potential synthesis in different subcellular compartments—i.e., mitochondria and chloroplasts—or the number of different sites described have been used as strong arguments to endorse membrane diffusion as the major mechanism of release. Yet, few experimental demonstrations have been found within the scientific literature.

Using optical absorption after dialysis, Lamy-Freund’s group studied the interaction of the indole with lipid bilayers, determining that melatonin crosses asolectin vesicles [120] and, using fluorescence and electron spin resonance spectroscopy (ESR), they further showed association between melatonin and lipids [121]. Nonetheless, rather than crossing, these and other studies have shown that the indole preferentially associates with the polar head groups [122,123], thus creating a sort of melatonin-rich membrane domain, particularly if the concentration used is high [124]. Since much of the melatonin would be tightly associated to the polar region, the speed for crossing a multilayer membrane would be low [122]. This has also been corroborated with in vivo studies by Venegas and colleagues [125], who reported melatonin accumulation in membranes, cytosol, mitochondria and nuclei. However, while an increasing dose led a 10-fold increase in membranes, mitochondrial and nuclear levels reached saturation, indicating that passive diffusion would not be the exclusive mechanism of melatonin transport. The presence of high concentrations of melatonin within or tightly associated to the lipid bilayer might explain the efficiency of the indole in reducing lipid peroxidation and preserving membrane fluidity [126,127,128]. Contrary to these studies, Yu and co-workers, using direct amperometric measurements, have shown the efflux of melatonin from human embryonic kidney cells, reaching a fast equilibrium at both sides of membranes [129]. It can be deduced from this bulk of data that more research is needed to clarify the real behaviour of melatonin within the cell membranes, but it appears clear that the indole, as it occurs with steroids, might use different strategies to move across plasma (or organelle) membranes.

## 3. An Alternative View: Protein-Facilitated Transport

As mentioned above, melatonin can interact with lipid bilayers, although currently there is no consensus about how this interaction occurs. The question remains as to what potential alternative mechanisms exist for such a rather low passage through the bilayers are taking place. A potential melatonin uptake by cells is not a new concept. Forty years ago, Bubenik et al., using immunohistochemical detection of the indole in retina and Harderian glands, observed a great increase after melatonin application. Therefore, they suggested a potential uptake mechanism and/or receptors in these organs [130], which was further assessed by other studies. In this context, compounds with structural similarities to melatonin, such as tryptophan or serotonin (or other monoamines), can be translocated by different members of the solute carriers (SLCs) superfamily, including SLC3, SLC7, SLC16 and SLC36 for tryptophan or SLC6, SCL18 and SCL22 for serotonin/monoamines, among others. These carriers are present at either the plasma membrane or the membranes of organelles such as vesicles and mitochondria, as has been recently suggested for melatonin [131,132,133].

The classic view of melatonin behavior with lipid bilayers has nevertheless left only a few studies focused on protein-mediated carriers or active transporters, as most of them were centered on the role of melatonin membrane receptors. However, the situation recently changed when Hevia and colleagues [134] challenged the classical idea by studying melatonin uptake in normal as well as in cancer cells. Using a specifically developed HPLC method [37] and a highly accurate estimation of the cell volume [135], these authors reported that both intracellular concentrations and kinetics adjust to a transporter-assisted mechanism rather than to a simple passive diffusion, a finding that was consolidated by studying different cell lines. Accordingly, no equilibration at both membrane sides was observed, contrary to what had been suggested by others [120], thus demonstrating that the indole might cross membranes through facilitated transport and not exclusively by passive diffusion. The carrier involved was later attributed to a member of the SCL2A/GLUT transporters subfamily [136]. The classic glucose transporter GLUT1/SLC2A1 would be the prototype member of this subfamily. However, it has only been very recently that the X-ray crystallographic structure of human GLUT1 has been elucidated [137] and until now the homologue XylE bacterial transporter has been employed as the reference protein model. With both docking models, it was predicted that melatonin binding was thermodynamically favorable. Moreover, as a physiological approach, the assays performed on melatonin uptake by erythrocytes, which only express GLUT1, showed that indole uptake was greatly enhanced by adding glucose to the growth medium [136], as it could be deduced if GLUT1 played a role in such internalization. Furthermore, overexpression of GLUT1, but not structurally-related GLUT4, leads to an increase in melatonin uptake in prostate cancer cells (Hevia et al., personal communication). Even though many other cell types and SLC2A carriers should also be investigated, this research field should provide additional data that may be of physiological importance.

Interestingly, melatonin has not been the only metabolite associated to GLUT1. It is well documented that, in addition to glucose, GLUT1/SLC2A1 is also in charge of transporting the oxidized form of ascorbic acid, i.e., DHA, into cells [138]. This might be the main role of GLUT1 in the erythrocytes of those species unable to synthesize vitamin C, a function that requires stomatin as a regulatory partner [139]. By similarities, it could be deduced that melatonin uptake through GLUT1/SLC2A1 transporter might also involve additional association with other regulatory factors. Finally, it is noteworthy to mention that the involvement of GLUT/SLC2A transporters in melatonin membrane transport can provide additional insights underlying the specific relationship between melatonin and glucose. However, it is not known whether it is GLUT1 itself or rather its association to other membrane proteins (e.g., membrane receptors) that would deserve further attention.

GLUT1 has not been the only transporter recently linked to melatonin membrane translocation. Similarly, Huo et al. [140] have described the possible involvement of PEPT1/2 oligopeptide transporters (proton-coupled SCL15A1/2 family) in the uptake of melatonin and its sulfation derivatives. SLC15A1 displays two isoforms in the rat; interestingly, while one is highly expressed in the small intestine, mainly participating in the absorption of protein digestion products [141], isoform 2 is restricted to pinealocytes, where it exhibits a striking circadian rhythmicity in its expression, with a clear 100-fold upregulation during the dark phase, which might underlie into the mechanism of melatonin secretion [142]. The study also reported the localization of PEPT1 in the mitochondria, therefore shedding some light on melatonin´s translocation into this organelle, although no transition peptide for directing this protein to mitochondria was described. Again, as it occurs with previous work, authors here showed a kinetics for the uptake that is incompatible with passive diffusion [140]. So, the questions that remain to be answered are whether these two transport systems, as it appears, can be compatible with each other and whether facilitated transport would provide a faster and, perhaps under specific physiological circumstances, an additional way to internalize melatonin into cells and/or organelles. The wide expression if any of these two transporters provide an answer to the pleiotropic role of melatonin in different cell types and tissues/organs, including metabolic and glucose-related effects.

## 4. Melatonin and Glucose: An Ancient Relationship?

### 4.1. Glucose Effect on Melatonin Secretion

Milcou and coworkers reported a relationship between the pineal and glucose homeostasis [143], but these pioneering studies used pineal extracts, since melatonin had not been yet isolated. Years later, one of the first associations between melatonin and diet glucose was made by Wetterberg’s laboratory. First, using 13 participants, his group showed that water-supplemented, short-term (2 days) fasting led to a significant decrease in nocturnal melatonin levels, an effect prevented by glucose intake during the fasting period [144]. This is similar to what occurred to the inhibition of the pituitary-testicular axis under the same type of fasting [145]. Authors tried to explain this reduction based on the dependence of glucose delivery on pinealocytes to function properly. Later on, the same group found that obesity does not alter the pattern of melatonin secretion [146]. Interestingly, other early evidence found that glucose itself may affect AANAT activity [143,147] but in some of these pioneering studies they used pineal extracts, since melatonin had not been yet isolated. Collectively, all this early evidence point out that glucose could modulate melatonin secretion. 

As described below, the antagonism between melatonin and insulin is well documented. But beyond those regulatory effects of the indole on insulin secretion, it has also been reported that streptozotocin-treated, diabetic rats display an elevated AANAT activity and consequently an increase in melatonin levels in the pineal gland [148]. Contrary to this, AANAT activity and melatonin content in the retina are both reduced in the retina of streptozotocin-treated rats [149], but none of the studies approached the involvement of glucose levels on this effect. This is also the case in type 2 diabetes patients, who have reduced melatonin secretion [150], and also a reduced night pineal melatonin synthesis is observed in type 2 diabetic Goto-Kakizaki rats [150,151]. Similarly, reduced night melatonin production has also been found in women with metabolic syndrome [152]. Cano and co-workers have also found a reduced melatonin content in high-fat fed rats, concomitant with hyperglycemia [153]. When the melatonin rhythm is compared between fasted and hyperglycemic rats, there is a shift in the night-peak pattern [154]. Again, all these studies demonstrate a relation between glucose levels and melatonin secretion, but the molecular insights underlying these effects are still unclear.

### 4.2. Melatonin and Insulin 

Conversely, in addition to the glucose effects on melatonin secretion, the indole itself is involved in controlling glucose homeostasis [155] (reviewed by [156,157]). In fasted rats, a rhythm in plasma glucose has been well described, with a peak in glucose starting at the dark phase, similar to what occurs at dawn in humans, and this evidence persisted even in hyperglycemia or with a normal pattern of glucose feeding. Not surprisingly, a circadian rhythmicity was reported for insulin secretion in rats and also in human subjects, with a nadir at midnight and peaking between noon and 6 pm [154,158]. Bailey et al. and Gorray et al. [159,160] were the first groups to demonstrate that pinealectomy results in a significant increase in insulin secretion, an effect that was confirmed using pineal incubation media. Further studies from different laboratories showed that pinealectomy increases glucose levels as well as glucose intolerance in rats, an effect prevented by melatonin administration [161,162]. Overall, as described above, most authors, therefore, agree that a physiological antagonism between melatonin and insulin occurs. Reciprocally, increased insulin plays a role on melatonin secretion from the pineal gland but while most studies showed an inhibitory effect [163], a few reports have shown a stimulatory effect under some circumstances [155].

This physiological function of melatonin appears to be mainly mediated by membrane receptor signaling. These receptors are highly expressed in pancreatic islets [164] and both MTNR1A/B membrane receptors have been involved not only in regulating insulin production [165,166,167] but also in glucagon and somatostatin [40,168]. In vivo data were confirmed by using cell culture models, in which melatonin directly modulates insulin secretion from pancreatic β-cells, an action directly mediated by membrane receptors [169,170,171]. Interestingly, some polymorphisms, particularly in MTNR1B, have been associated with a higher risk in type 2 diabetes, thus reinforcing the role of this G-coupled membrane receptors on insulin synthesis regulation [172,173,174]. However, whether this is due as has been suggested to an excess of melatonin signaling [175] or rather, on the contrary, to a defective receptor function is still a matter of debate [176]. 

Other molecular mechanisms of action have also been related to the melatonin–insulin axis. Hence, recent studies point out the involvement of redox-related pathways, namely NADPH oxidase [177]. Similarly, the direct or indirect free radical-scavenging action of melatonin might also play a role in this context, as the indole protects glucotoxicity-mediated pancreatic islets cell death through either its own signaling or an antioxidant pathway [178,179,180]. More recently, insulin-like growth factor binding proteins, such as IGFBP3, which can modulate insulin signaling, have been linked to melatonin actions in other tissues [181] so they should not be ruled out in playing a role in melatonin-induced glucose homeostasis. 

All this strong evidence and compelling data have led some authors to propose melatonin as a potential therapeutic agent in diabetes [182,183], although the timing of administration might have different outcomes [184]. A summary of this evidence is shown in Table 3.

### 4.3. Melatonin and Glucose in Invertebrates and Protozoans.

Regulation of glucose metabolism by melatonin does not appear to be restricted to vertebrates. Most of the studies have been accomplished in crustaceans. Thus, in the crab *Eriocheir sinensis*, melatonin injection provoked hyperglycemia by inducing crustacean hyperglycemic hormone (CHH) mRNA synthesis and a similar effect was observed in *Uca pugilator* [190] and in *Neohelice granulata* [100], thus demonstrating an ancient relationship between melatonin and glucose.

Apart from invertebrates, even some protozoans with melatonin synthetic ability, e.g., *Tetrahymena*, were seen to respond to insulin in an autocrine mode, but the relationship between the indole and glucose metabolism has not yet been approached [191].

## 5. Concluding Remarks

There is no doubt that melatonin is a ubiquitously molecule, present in evolutionarily different organisms, from protozoa to mammals or higher plants. Although the pineal gland is responsible for the serum night peak, it is also synthesized in a variety of tissues and organs, therefore indicating a well-conserved function(s) in all living cells. Considering that glucose is one of the preferred sources for energy and carbon and the particular relationship that seems to be between both, melatonin-related glucose homeostasis might be one of the primitive functions of the indole. Here we have reviewed most of the data that link melatonin with glucose metabolism, including glucose control of melatonin synthesis, the physiological role of pineal melatonin in controlling insulin secretion, and finally novel findings relating GLUT1/SLC2A transporter and melatonin uptake, among others. 

However, there are still several questions that remain to be answered regarding melatonin and glucose: (i) is melatonin one of the major and important factors that control glucose metabolism in cells and tissues? (ii) Are membrane receptors the exclusive mediators of what seems to be a major melatonin-glucose interrelation? (iii) Could GLUT/SLC2A glucose transporters also mediate and control glucose homeostasis by interacting with the melatonin uptake? iv) How universal throughout the different phyla is the glucose homeostasis exerted by the indole? 

These questions urgently need an experimental approach, since they have multiple implications for evolutionary aspects, but also in pathologies with high incidence, such as diabetes and the metabolic syndrome.

## Figures and Tables

**Table 1:** Extra-pineal sites of melatonin production reported to date.

Organ/Tissue		Strategy	Original Reference(s)
**Retina, cerebellum**		Immunohistochemical localization of aaNAT	Bubenik et al. (1974) [27]; Quay (1983) [61].
**Gut (Enterochromaffin cells)**		Frog skin melanophores lightening of enterochromaffin cells extracts	Raikhlin et al. (1975) [28]; Raikhlin & Kvetnoy (1976) [62]
**Airway epithelium, adrenal, thyroid gland, liver, renal cortex, gallbladder, inner ear, ovary, endometrium, placenta, mast cells, NK cells, eosinophils, thymus**		Immunohistochemistry	Raikhlin et al. (1975) [28]; Raikhlin & Kvetnoy (1994) [63]; Kvetnoy et al. (2001) [64]
**Harderian gland**		NAT/ASMT enzymatic activities, immunohistochemical localization, RIA, NAT and ASMT enzymatic activities, immunohistochemistry of melatonin	Cardinali & Wurtman (1972) [26]; Bubenik et al. (1976a y 1976b) [29,65]; Pang et al. (1977) [66]; Menéndez-Peláez et al. (1987) [67]; Bubenik, G.A. (1980) [68]
**Digestive tract**			
**Cochlea (inner ear)**		RIA, TLC (detection of radio-labelled metabolites after primary culture in medium supplemented with [^14^C]5-HT) Direct enzyme-linked immunosorbent assay	Biesalski et al. (1988) [69]; López-González et al. (1997) [70]
**Peripheral blood mononuclear cell (PBMCs)**		HPLC, TLC, Mass Spectrometry, RIA	Finocchiaro et al. (1991) [71]
**Eye**	**Ciliary body**	HPLC (electrochemical detection), NAT/ASMT enzymatic activities	Martin et al. (1992) [71]
	**Crystalline**	RIA, HPLC (for precursors), NAT/ASMT enzymatic activities	Abe et al. (1999) [72]
**Skin**		HPLC (fluorometric detection), Mass spectrometry, NAT/ASMT activity and expression (RT-PCR)	Slominski et al. (1996; 2002) [73,74]
**Testis**		TLC, NAT/ASMT enzymatic activities	Tijmes et al. (1996) [75]
**Ovary**		HPLC (fluorometric detection), RIA, NAT/ASMT enzymatic activities	Itoh et al. (1997; 1999) [76,77].
**Bone marrow**		RIA, HPLC (electrochemical detection), Mass spectrometry Immunocytochemistry, NAT enzymatic activity, ASMT expression	Tan et al. (1999) [78]; Conti et al. (2000) [79]
**Thymus, spleen, lung, heart, kidney, muscle, liver, stomach, gut, testis, spinal cord, brain, platelets**		NAT/ASMT expression	Stefulj et al. (2001) [79]
**Thymus**		HPLC (fluorometric detection), NAT/ASMT enzymatic activities	Jiménez-Jorge et al. (2005) [79]
**Lymphocytes**		HPLC (fluorometric detection), NAT/ASMT activity and expression	Carrillo-Vico et al. (2004) [80]
**Placenta**		NAT/ASMT expression (RT-PCR)	Iwasaki et al. (2005) [81]
**Liver, kidney, heart**		ELISA, NAT/ASMT expression (RT-PCR)	Sánchez-Hidalgo et al. (2009) [81]
**Mast cells**		ELISA, NAT/ASMT activity and expression (RT-PCR)	Maldonado et al. (2010) [82]

**Table 2:** Melatonin in microorganisms and invertebrates.

Organism	Strategy for Detection	Original Reference(s)
**EUBACTERIA**		
***Rhodospirilluym rubrum***	RIA	Manchester et al. (1995) [45]
*Erythrobacter longus*	RIA	Tilden et al. (1997) [83]
*Bacillus* sp.	UPLC-MS/MS	Jiao et al. (2016) [84]
*Euglena gracilis*	HPLC	Balzer et al. (1996) [85]
**PROTISTS**		
**Lingulodinium (syn Gonyaulax) polyedra (Dinoflagellate)**	HPLC	Poeggeler and Hardeland (1994) [86]
**Saccharomyces cerevisiae (Yeast)**	HPLC	Sprenger et al. (1999) [87]
***Euglena gracilis***	HPLC	Pandi-Perumal and Cardinali (2007) [87]
***Trypanosoma cruzi***	RIA	Macías et al. (1999) [47]
Other Dinoflagellates including: *Alexandrium* (sp.), *Ceratium horridum*, *Amphidinium carterae*, *Pyrocystis lunula*, *Noctiluca scintillans*	HPLC/RIA	Data obtained from abstracts or proceedings
Ciliates: *Tetrahymena thermophila*	HPLC	Kohidai et al. (2003) [88]
**LOWER INVERTEBRATES**		
***Dugesia japonica***	Biosynthetic enzymes	Itoh et al. (1999) [89]
**Caenorhabditis elegans**	Biosynthetic enzymes	Migliori et al. (2012) [90]
***Lumbricus terrestris***	Spectrophotometry	Subaraja and Vanisree (2016) [91]
**ARTHROPODA**		
*Locusta migratoria*	RIA	Vivien-Roels et al(1984) [49]
*Drosophila melanogaster*, *Periplaneta americana*	TLC	Finocchiaro et al. (1988) [92]; Richter et al. (2000) [93]
*Musa autumnalis*	RIA	Wetterberg et al. (1987) [94]
*Daphnia magna*	ELISA/IHC	Markowska et al. (2009) [95]
Decapoda: *Carcinus maenas*, *Uca pugilator*, *Nephrops norvegicus*, *Procambarus* sp., *Neohelice granulat*, *Eriocheir sinensis*	RIA/HPLC	Vivien-Roels and Pevet (1993) [96]; Tilden et al. (1997) [97]; Aguzzi et al. (2009) [98]; Farca-Luna et al. (2010) [99]; Maciel et al. (2014) [100]; Yang et al. (2018) [101]
**COELENTERATES**		
***Renilla koellikeri***	RIA	Mechawar and Anctil (1997) [102]
**MOLLUSCA**		
***Aplysia californica***	HPLC	Abran et al. (1994) [103]
***Sepia officinalis***	RIA	Vivien-Roels and Pevet (1986) [52]

**Table 3:** Summary of the evidences/links between glucose or insulin and melatonin.

Evidence/Finding Reported	Type of Assay/Molecular Mechanism Demonstrated	Reference(s)
**Glucose Influence on Melatonin Secretion**		
**Short-term fasting inhibits melatonin secretion (*H. sapiens*)**	N/A	Röjdmark & Wetterberg (1989) [144]
**Glucose affects AANAT activity (*R. norvegicus*)**	Enzyme activity	Welker & Vollrath (1984) [147]
**Streptozotocin increases pineal AANAT (*R. norvegicus*)**	Enzyme activity	Peschke et al. (2008) [148]
**Streptozotocin decreases retinal AANAT (*R. norvegicus*)**	Enzyme activity, melatonin content (RIA)	Buonfiglio et al. (2011) [149]
**Type 2 diabetes patients display reduced night melatonin**	Melatonin content (RIA); urine 6-sulfatoxymelatonin (RIA)	Peschke et al. (2006) [150]; McMullan et al. (2013) [150]
**Type 2 diabetic Goto-Kakizaki rats display reduced night pineal melatonin**	Plasma and pineal melatonin and precursors content (RIA)	Peschke et al. (2006) [150]; Frese et al. (2009 ) [151]
**Metabolic syndrome women show lower nigh melatonin**	Melatonin content (RIA)	Corbalán-Tutau et al. (2014) [152]
**Hyperglycemia (high fat diet, *R. norvegicus*) shifts melatonin peak**	Melatonin content (RIA)	Cano et al. (2008) [153]
**Induced diabetes reduces night pineal melatonin content (*R. norvegicus*)**	Melatonin content (RIA)	Champney et al. (1983) [163]
**Melatonin and Insulin Secretion**		
**Circadian rhythm in insulin secretion (*R. norvegicus, H. sapiens*)**	RIA, Immunoreaction	Rigas et al. (1968) [185]; Gagliardino y Henández (1971) [186]; Boden et al. (1996) [187]
**Correlation between melatonin and insulin levels**	RIA	Bizot-Spinard et al. (1998) [154]; Peschke et al. (2013) [155]
**Pinealectomy-increase in insulin secretion**	N/A	Bailey et al. (1974) [159]; Gorray et al. (1979) [160]
**Pinealectomy increases glucose intolerance**	Immunoreaction	Díaz y Blázquez (1986) [161]
**Presence of melatonin receptors in pancreatic cells**	Western blotting/IHC	Nagorny et al. (2011) [164]; Zibolka et al. (2018) [188]
**Melatonin inhibits insulin secretion from pancreatic beta-cells**	MTNR1B receptor-mediated/cGMP Raf-1/ERK mediated/NADPH oxidase	Stumpf et al. (2008) [169]; Mühlbauer et al. (2011) [165]; Li et al. (2018) [171]; Simoes et al. (2016) [177]
**Melatonin influences somatostatin and glucagon**	MTNR1A/B receptor-mediated	Bähr et al. (2011) [40]; Zibolka et al. (2015) [168]
**Melatonin protects against glucotoxicity**	Prevents ER stress	Park et al. 2014 [178]
**MTNR1 polymorphisms and type 2 diabetes association**	Genetic polimorphisms/altered MTNR1 signalling pathway	Bouatia-Naji et al. (2009) [189]; Sparso et al. 2009 [174]; Mssig et al. (2010) [172]; Tam et al. (2010) [173]; Tuomi et al. 2016 [175]; Mulder 2017 [176]
